# Role of warm ischemia on innate and adaptive responses in a preclinical renal auto-transplanted porcine model

**DOI:** 10.1186/1479-5876-11-129

**Published:** 2013-05-24

**Authors:** Ludivine Rossard, Frédéric Favreau, Sebastien Giraud, Raphael Thuillier, Sylvain Le Pape, Jean Michel Goujon, Alexandre Valagier, Thierry Hauet

**Affiliations:** 1 Inserm U1082, Poitiers, F 86000, France; 2 Université de Poitiers, Faculté de Médecine et de Pharmacie, Poitiers, F 86000, France; 3 CHU de Poitiers, Service de Biochimie, Poitiers, F 86000, France; 4 INRA, IBISA, Domaine expérimental du Magneraud, Surgères F 17700, France

**Keywords:** Ischemia-reperfusion, Adaptive immune response, Innate immune response, Kidney transplantation, Preclinical porcine model

## Abstract

**Background:**

Deceased after cardiac arrest donor are an additional source of kidney graft to overcome graft shortage. Deciphering the respective role of renal warm and cold ischemia is of pivotal interest in the transplantation process.

**Methods:**

Using a preclinical pig model of renal auto-transplantation, we investigated the consequences of warm and cold ischemia on early innate and adaptive responses as well as graft outcome. Kidneys were subjected to either 60 min-warm ischemia (WI) or auto-transplanted after cold storage for 24 h at 4°C (CS), or both conditions combined (WI + CS). Renal function, immune response and cytokine expression, oxidative stress and cell death were investigated at 3 h, 3 and 7 days (H3, D3 and D7) after reperfusion. At 3 months, we focused on cell infiltration and tissue remodelling.

**Results:**

WI + CS induced a delayed graft function linked to higher tubular damage. Innate response occurred at D3 associated to a pro-oxidative milieu with a level dependent on the severity of ischemic injury whereas adaptive immune response occurred only at D7 mainly due to CS injuries and aggravated by WI. Graft cellular death was an early event detected at H3 and seems to be one of the first ischemia reperfusion injuries. These early injuries affect graft outcome on renal function, cells infiltration and fibrosis development.

**Conclusions:**

The results indicate that the severe ischemic insult found in kidneys from deceased after cardiac arrest donor affects kidney outcome and promotes an uncontrolled deleterious innate and adaptive response not inhibited 3 months after reperfusion.

## Background

In organ transplantation, graft shortage increases morbidity on dialysis; prolongs waiting times for adults and urges transplant physicians to accept new source of organs. Donor deceased after cardiac death (DCD), is an additional potential source of kidney graft which is more prone to severe ischemia reperfusion injury (IRI), primary non-function (PNF), and delayed graft function (DGF). In such donors, different ischemic conditions are associated such as warm ischemia (WI) and cold ischemia (CS) characterized respectively by an ischemic period at body temperature initiated by cardiac arrest ensuing a low and/or no blood flow and by a hypothermic preservation of kidneys. However, the respective role of WI and CS in transplantation conditions needs to be clarified to improve the use of graft from DCD, particularly from uncontrolled donors (class I, II and V of Maastricht classification). Consequently, researches into the mechanisms of WI, CS, and IRI are necessary to maximise the use of available donor pool and to minimise the PNF and DGF.

We and other have previously demonstrated that ischemia reperfusion sequence involved in renal auto-transplantation model induces inflammatory processes that are independent of the presence of allo-antigen [1,2] and characterized by both innate and adaptive immune responses [2-4]. The pathophysiology of renal transplantation process involves a complex interplay among vascular, tubular, and inflammatory factors followed by a repair process or progressive fibrotic chronic kidney disease when it persists. The complex interplay between innate and adaptive immunity is still not completely understood and the chronological response related to the severity of graft lesions needs to be clarified. There is also a strong body of evidence that the consequences of IRI are identifiable in an increased acute rejection rate [5,6].

In ischemic research, most experiments are carried out on rodents, but crucial prerequisites for the development of safe clinical protocols are needed through suitable large animal models like pig [7,8]. Because the renal porcine anatomy and vascular bed are very similar with human, we used a porcine autologous renal transplant model. In addition, such model is an ideal situation for tolerance and allows focusing on IRI effects *per se*.

We hypothesize that the ischemic period is a crucial time point which is involved in the initial development of chronic graft injuries observed in the first months of reperfusion and that WI before transplantation induces an uncontrolled response which promotes a worst graft outcome. Thus, we propose to decipher the respective influences of CS and WI in a preclinical model of kidney transplantation on innate and adaptive response with a high degree of translation to the clinic, in an attempt to determine the chronology of lesion development and find the most discriminating markers and time windows to evaluate graft quality.

To this end, we have developed and validated different models to decipher WI and cold preservation injuries and the combined effect of these conditions enabling a step by step study [9-13]. The goal of the present work was to study the modulation of an ischemic preclinical model combining 60 min WI and 24 h CS followed by transplantation mimicking different clinical situations found in uncontrolled donors and particularly to decipher the innate and adaptive responses between WI and CS to easily manage the pharmacological approach.

## Methods

### Surgical procedure and experimental groups

We used a well controlled model of large white male pigs weighting 30 to 35 kg [2]. The surgical and experimental protocols were performed in accordance with the institutional committee for the use and care of laboratory animals: COMETHEA (CEEA Poitou-Charentes, project reference numbers: CE2012-4 and CE2012-29). We determined 3 experimental conditions deciphering WI, CS and combination of WI and CS: 1. WI: 60 min *in vivo* ischemia by renal pedicle clamping without transplantation to mimic clinical situation of no-reflow (n = 12); 2. CS: kidney was removed, cold flushed, preserved at 4°C for 24 h in UW and autotransplanted (n = 12); 3. WI + CS: kidneys were subjected to both conditions to mimic DCD (n = 12). In each experimental group, the controlateral kidney was removed to mimic the graft nephron mass found in clinical situation. Reperfusion was studied at: 3 hours (H3), 3 and 7 days (D3 and D7), and 3 months (M3) with pig sacrifice at each time point. Native kidney is also used as control group.

### Renal function determination

Urinary and plasma sodium for fractional excretion of sodium (FeNa^+^) evaluation were determined at H0, H3, D3 and D7. Plasma creatinin was measured at H0, H3, D3 and D7 and M3 after reperfusion and urinary protein excretion was determined at M3 after reperfusion using an automated chemistry analyzer (Modular, Roche Diagnostics, France).

### Histochemical and immunohistochemical study

Histochemistry for tubular atrophy and Red Sirius staining on cortex samples was performed as previously described [2]. Immunohistochemical studies were performed using anti-CD3 (Cell marque Corporation, USA), and anti-ED1 (AbD Serotec, USA). All sections were examined under blind conditions by a pathologist. Apoptotic signals were characterized by immunostaining using anti-cleaved caspase 3 antibodies (R&D System, USA).

### Real-time quantitative PCR

We used RNA extraction kit (Qiagen, France). Genomic DNA was removed using DNA-free kit (Applied Biosystems, USA) and first-strand reverse transcription was performed. Real-Time PCR assays were performed on an ABI Prism 7300 (Applied Biosystems) with porcine primers (Additional file 1: Table S1).

### Western blot analysis

Western Blotting was carried out according to standard protocols [14] using specific antibodies against superoxide dismutase-1, transforming growth factor β, pro-matrix metalloproteinase-2, tissue plasminogen activator (SOD-1, TGFβ, pro-MMP-2, tPa, Santa Cruz, USA), pSmad3 (pSmad3, Cell signalling, France), monocyte chemoattractive protein-1 (MCP-1, Clinisciences, France), bone morphogenetic protein-7 (BMP-7, AbD Serotec, France), plasminogen activator inhibitor-1 (PAI-1, BD-Bioscience, USA) and loading control: glyceraldehyde 3-phosphate dehydrogenase or β actin (GAPDH, βactin, Sigma Aldrich, France).

### Statistical analysis

Values are reported as the mean ± SEM. Statistical differences between groups were calculated using ANOVA followed by the Student-Newman-Keuls test or Kruskal Wallis test for multiple comparison analysis. Correlation analysis was performed with NCSS software (NCSS LLC, USA), r^2^ is displayed and significance established with the Spearman rank test. p < 0.05 was considered as significant.

## Results

### Severe IRI affects early renal function in preclinical model

WI increased CS-induced delayed graft function recovery and chronic dysfunction. A significant increase in creatininemia was observed in experimental groups subjected to ischemia from H3 maintained at D3, with the highest level attained in WI + CS (Table 1). Although independently, WI and CS groups partially restored their renal function at D7, whereas WI + CS creatinin levels were still elevated. FeNa^+^ showed poor tubular function in CS group, compared to some dysfunctions in WI and highest FeNa^+^ in WI + CS group (Table 1). At M3, a significant increase in creatininemia was observed in ischemic groups, with the highest level reached in WI + CS, followed by CS alone (Table 1). Proteinuria showed the same pattern (Table 1).

**Table 1:** Renal function evaluation by systemic and urinary parameters levels before, at H3, D3 , D7 and M3 after reperfusion in warm ischemia (WI), cold storage (CS), warm ischemia + cold storage (WI + CS) groups

**Creatininemia (μmol/L)**	**D0**	**H3**	**D3**	**D7**	** M3**
WI group	88 ± 5	142 ± 3	435 ± 3	180 ± 8	122 ± 2
CS group	86 ± 5	337 ± 5^**‡**^	1031 ± 34^**‡**^	169 ± 8	147 ± 4^**‡**^
WI + CS group	85 ± 2	412 ± 10^**‡**^ º	1823 ± 108 ^**‡**^ º	447 ± 70 ^**‡**^ º	335 ± 15^**‡**^ º
**Fractional excretion of sodium (FeNa+,%)**	**D0**	**H3**	**D3**	**D7**	
WI group	0.18 ± 0.01	ND	5.79 ± 0.38	2.93 ± 0.22	
CS group	0.22 ± 0.02	ND	18.67 ± 1.20‡	7.29 ± 0.31‡	
WI + CS group	0.17 ± 0.02	ND	ND	12.73 ± 0.21‡º	
**Proteinuria (g/L)**	**M3**				
WI group	0.50 ± 0.15				
CS group	2.80 ± 0.50 ‡				
WI + CS group	4.10 ± 0.80 ‡ º				

Mean ± SEM, ‡ p < 0.05 vs WI, and º p < 0.05 vs CS group, *ND* No diuresis.

### Severe ischemia induces a pro-oxidative milieu and cell death

**Figure 1:**
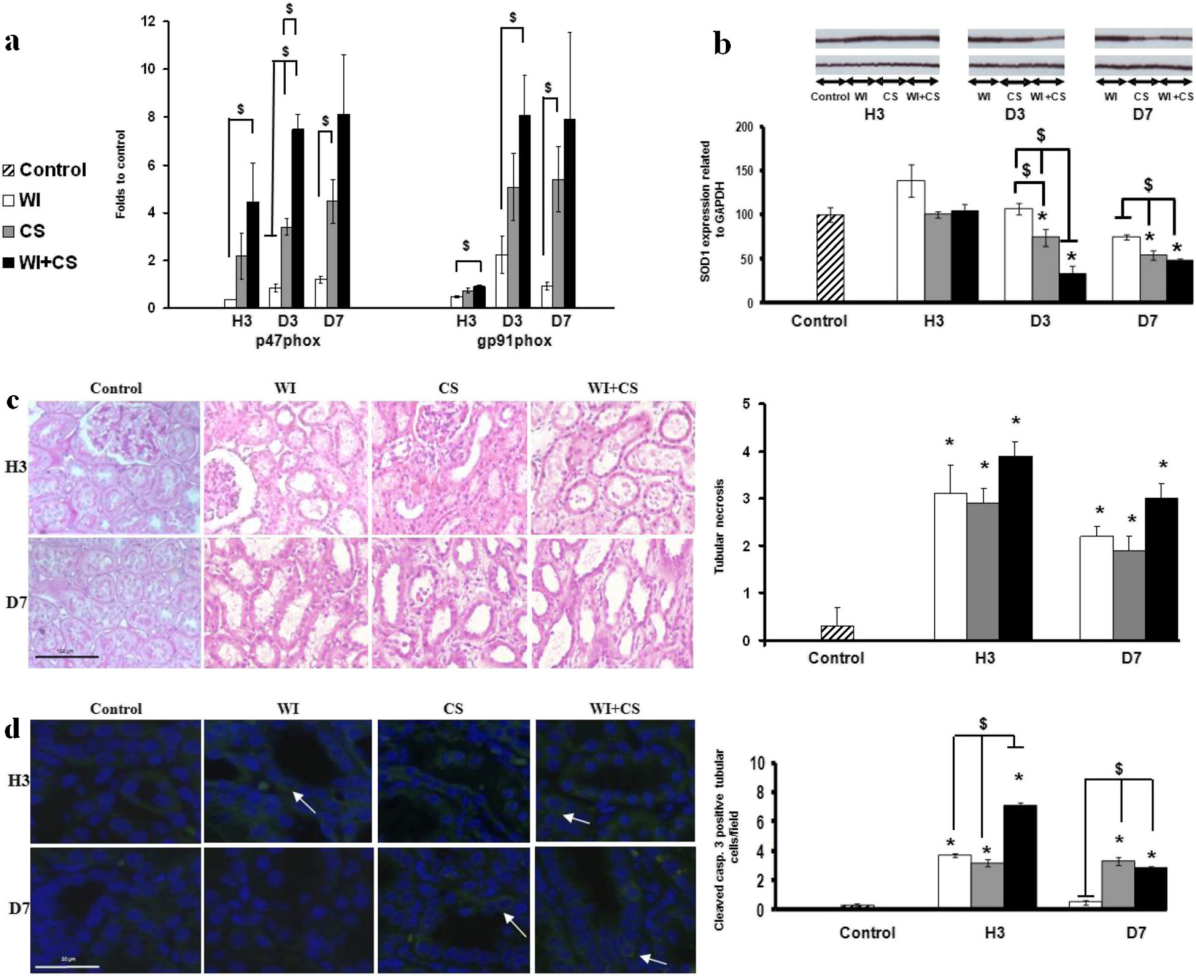
**Intensive ischemic injury induces early pro oxidative milieu.** The mRNA expressions of p47phox and gp91phox (**a**), and protein expression of cytoplasmic Cu/Zn SOD (SOD1, **b**) were investigated in control, WI, CS and WI + CS groups. **Ischemia induces early necrosis and apoptosis.** The numbers of necrotic tubular cells (**c**) and positively cleaved caspase 3 tubular cells (**d**) per field were counted on ten different tissue sections for each experimental condition in the first week of reperfusion. Shown are mean ± SEM. *p < 0.05 versus control. $ p < 0.05 between groups.

Nicotinamide adenine dinucleotide phosphate oxidase (NADPH oxidase) is an important generator of superoxide anion during inflammatory processes mainly expressed by endothelial cells and neutrophils [15]. We investigated the mRNA expression of two NADPH oxidase subunits: gp91phox and p47phox (Figure 1a). While WI did not induce an up regulation, cold preserved grafts exhibited a higher expression from D3 related to the intensity of injury. In contrast to gp91ph*ox* subunit, the p47phox seemed to be early expressed at H3 in the severe ischemic group WI + CS (Figure 1a). The decrease of SOD1 protein expression, a major antioxidant enzyme, was significant at D3 and D7 in CS group and more pronounced in WI + CS group at D3 (Figure 1b).

The apoptotic and necrotic processes are early associated with ischemic conditions. Tubular necrosis was early observed in all studied group at H3 and maintained at D7 compared to control (Figure 1c). The WI + CS group was characterized by early high cleaved caspase 3 staining; less marked in WI and CS groups (Figure 1d). At D7, CS and WI + CS groups exhibited a high expression of cleaved caspase 3 in contrast to the basal values observed in WI group (Figure 1d).

### Early innate and adaptive immunity depend on the severity of IRI

**Figure 2:**
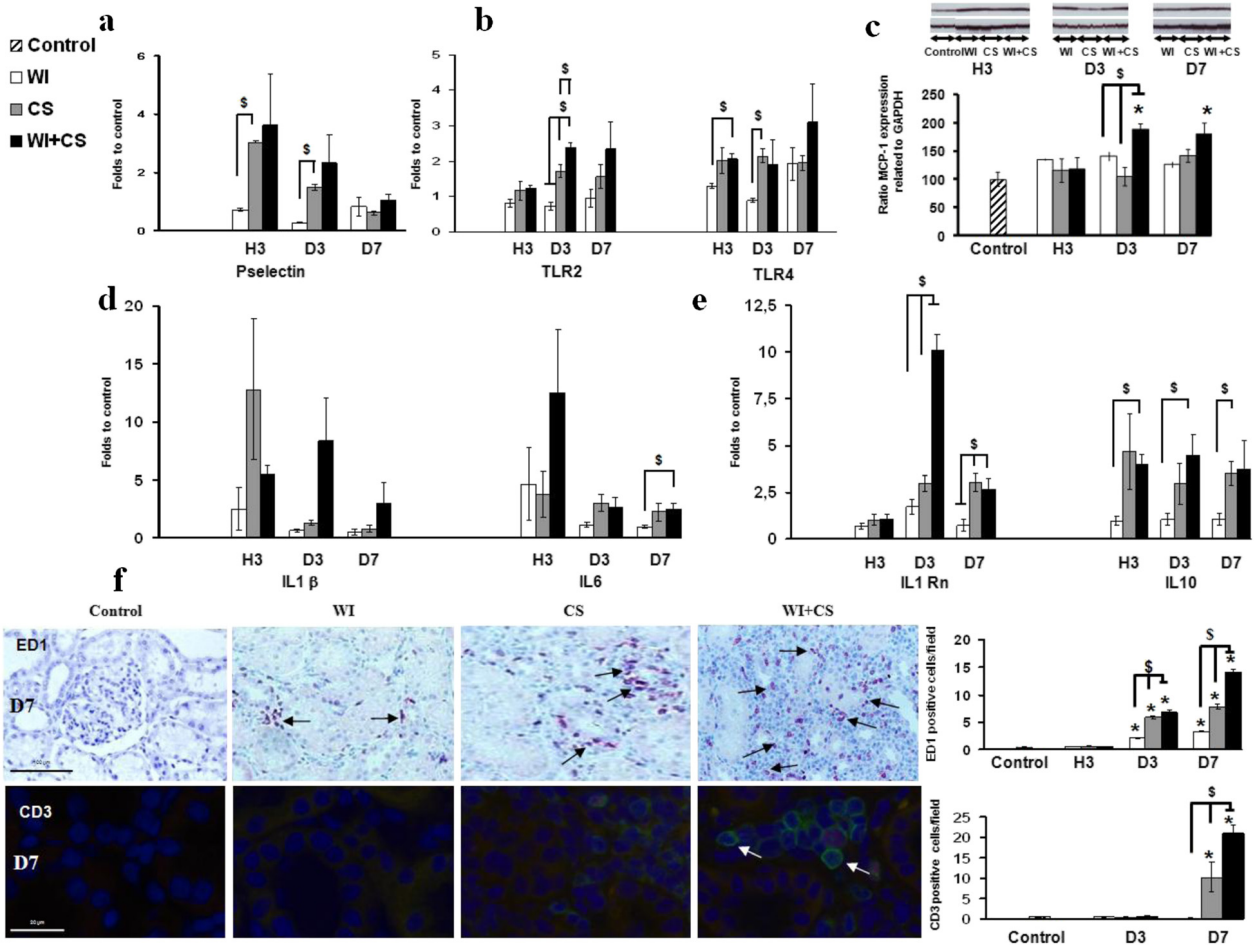
**Chronological analysis of early immune process in reperfused kidneys.** The P-selectin mRNA was studied as marker of endothelial activation (**a**). Innate immunity markers like Toll-like receptors 2 (TLR2) and TLR4 mRNA (**b**), MCP-1 protein (**c**), IL-1β and IL-6 cytokines mRNA (**d**) were investigated in kidney tissue. IL-1Rn and IL-10 cytokine mRNA expressions were also explored (**e**). The numbers of positively ED1 and CD3 stained cells per surface area (10^4^/μm^2^) was counted on five different tissue sections for each experimental conditions (**f**). Shown are mean ± SEM. *p < 0.05 versus control. $ p < 0.05 between groups.

P-selectin was temporary expressed in cold ischemia groups at H3 and D3 supporting an early and transient endothelial activation after cold ischemia (Figure 2a). Toll-like receptors (TLRs) and particularly TLR2 and 4 are central in the early activation of the innate immune response in the setting of IRI [16,17]. TLR4 mRNA expression was early increased at H3, to similarly high levels in CS and WI + CS, while remaining low in WI (Figure 2b). TLR2 mRNA was up-regulated in CS at D3 and higher in WI + CS, a trend was maintained at D7 (Figure 2b). In our study, MCP-1 protein expression was increased from D3 to D7 in WI + CS group (Figure 2c). IL1β was up-regulated early (H3, Figure 2d), but only WI + CS grafts showed maintained expression at D3 and D7. IL6 was similarly up-regulated (Figure 2d). High mRNA expression of IL-1Rn, the interleukin-1 receptor antagonist (IL-1RA), was detected in WI + CS grafts at D3 (Figure 2e). Expression remained above control values at D7 but was not discriminating between experimental groups exposed to CS condition. Warm ischemic kidneys did not show up regulation of IL-1Rn expression. IL-10 expression was also absent in WI group (Figure 2e), while CS and WI + CS grafts showed consistent overexpression at different studied times of reperfusion.

ED1+ cells infiltration started early at D3 with higher intensity in CS and WI + CS. At D7, this process indicated potential macrophages phagocytosis gradually developing in relation with the severity of IRI [18,19] (Figure 2f). In turn, a drastic CD3+ lymphocyte invasion was recorded in CS groups with a higher degree in WI + CS group only at D7 in contrast to baseline level in WI (Figure 2f).

### Warm ischemia increases cold storage-induced immune response, tubular injury and fibrotic response in the long term

**Figure 3:**
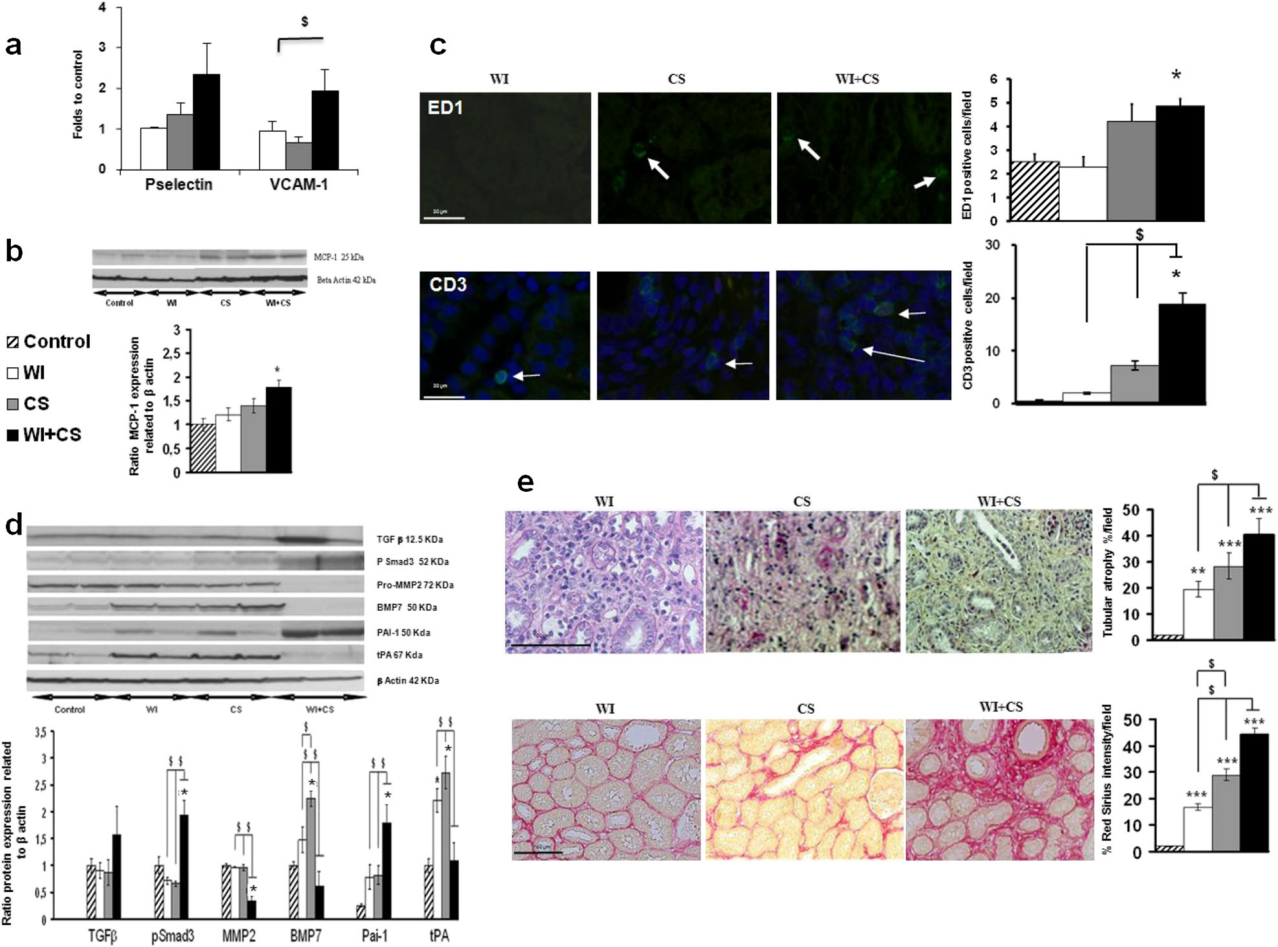
**WI + CS maintained inflammatory cells recruitment 3 months after reperfusion and induced tubular injury and fibrosis.** VCAM-1 and P-selectin mRNA and MCP-1 protein expressions were investigated in each studied conditions (**a-b**).The numbers of positively ED1 and CD3 (**c**) stained cells per surface area (10^4^/μm^2^) was counted on ten different tissue sections for each experimental conditions. Tubular atrophy, Red Sirius staining (**e**), and protein expression (**d**) of mediators of TGF beta and tPA pathways were studied in control, WI, CS and WI + CS groups at 3 months post-reperfusion. * p < 0.05 vs. control, $ p < 0.05 between ischemic groups.

At 3 months after reperfusion, WI + CS maintained a high recruitment of ED1+ cells in renal tissue (Figure 3c). These results were supported by VCAM-1 and MCP-1 expressions (Figure 3a-b). Lymphocytes recruitment was also preserved in WI + CS group (Figure 3c). In addition, a single clamp of renal artery induced an important tubular atrophy accentuated in CS condition, and upgraded when WI is combined to CS (Figure 3e). This response was associated with extracellular matrix and collagen production, with a severe grading in the WI + CS condition up to 45% (Figure 3e). This response was supported by pro-fibrotic pathways activation (Figure 3d). Indeed, TGFβ expression tends towards increased in WI + CS group supported by significant up-regulation of its downstream mediator pSmad3 and down-expression of its inhibitor BMP-7. In fact, CS group exhibited an increased expression of BMP-7 which failed when WI was combined. The same pattern was observed with tPA which could be attenuated by PAI-1. Indeed, we observed an up-regulation of PAI-1 in WI + CS well known to inhibited tPA and favored renal extracellular matrix deposition accentuated by a decrease of MMP-2 expression.

### Adaptive response affects early and chronic graft function

**Figure 4:**
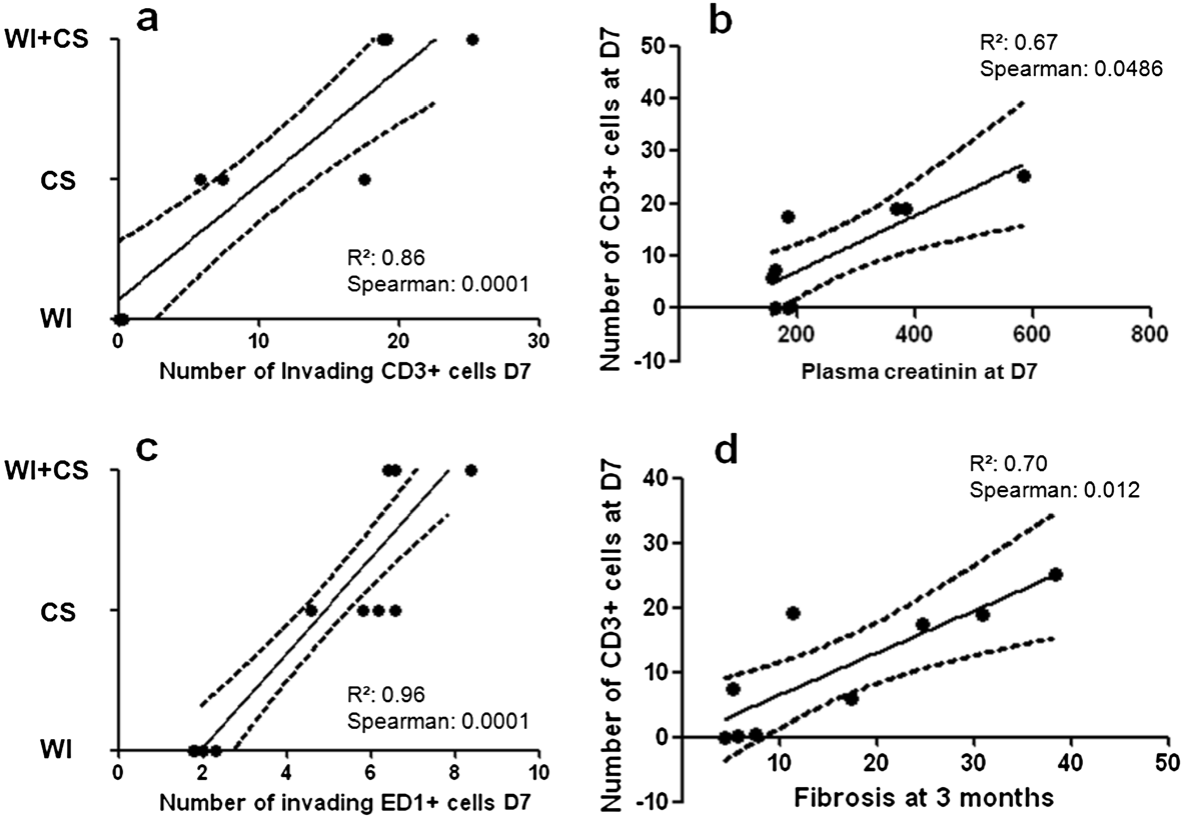
**Adaptive response affects early and chronic graft function.** Invading CD3+ and ED1+ cells in kidney graft correlate with the severity of ischemic injuries (**a**, **c**). The creatininemia after 7 days of reperfusion and fibrosis percentage evaluated at 3 months correlate with the number of lymphocytes invading the tissue at the end of the first week of reperfusion (**b**, **d**). r² and Spearman rank are indicated.

Although, the inflammatory process mediated by monocytes and lymphocytes recruitment is correlated with the severity of ischemic injuries induced by warm and cold ischemia and the combined conditions (Figure 4a-c), only the number of CD3+ cells invading the renal tissue is correlated with plasma creatinin at D7 and correlated with the intensity of collagen expression at 3 months (Figure 4b-d).

## Discussion

The worldwide shortage of standard brain dead donors has revived the use of kidneys from DCD. Therefore, with the required framework, DCD kidney transplant programs were reintroduced at the turn of the century. Experimental approach clearly defined that ischemic period is a crucial time which affects organ outcome [20]. Since 1994, we developed several pre-clinical pig models mimicking clinical situations. The aim of this study was to decipher the deleterious effects induced in organ from DCD conditions to improve and adapt graft preservation. A previous study described the effect of both WI and CS in *ex*-*vivo* porcine model without a separated analysis of each condition [21]. In addition in this report, the renal function was measured over a period of 3 hours. In our study, three types of IRI and their respective roles were compared: WI accompanied by no-reflow condition (shock, cardiac or aortic surgery or asystolic time) and CS involved in transplantation process, potentially preceded by WI in DCD conditions. In these conditions, the inflammatory and immune response which are key processes involved in repair as well as in chronic kidney fibrosis were studied at 3 hr, 3 days and 7 days for early stage after reperfusion and also at 3 months [22].

During the first week of reperfusion, renal function in groups exposed to CS was more affected than in WI group. Our auto-transplant setting is an emblematic situation of tolerance, in which IRI *per se* can break this tolerance and destines the graft to a degraded chronic outcome. The initiation of immune response appears in the early hours in an identical fashion in all conditions; however it is maintained and will expand in proportion to the level of injury, supporting the concept of immunity is a solid discriminatory tool between damage intensities. Interestingly, we correlated the number of adaptive immune cells recruited with plasma creatinin levels at the end of the first week of reperfusion underlining a direct relationship between the intensity of inflammatory process and pejorative graft outcome.

Oxidative stress and apoptosis are two major components of IRI, both closely associated to the inflammatory process [23]. We demonstrated that CS combined or not with WI induced an overexpression of NADPH oxidase enzyme subunits during the first week of reperfusion in contrast to WI alone. This complex may participate in the superoxide anion production by inflammatory cells infiltrating the graft, or directly from the injured tissue. This pro-oxidative milieu was accentuated by a decreased expression of the major antioxidant enzymes: SODs already reported in a rodent model [24]. These results indicate that severe ischemic conditions induce an early pro-oxidative microenvironment and that D3 is a critical time point to evaluate the red/ox balance. Apoptosis is also known to be an important response to ischemic injury [25]. Our results indicate an early activation of the apoptotic process few hours after ischemia. The high level of cleaved caspase 3 staining at H3 and the return to basal values at D7 in WI group suggests an early transitory apoptotic process. However, the high level of injury in WI + CS group at H3 was associated with a high staining level but limited in CS group. On the other hand, at D7 this expression was similar in both CS groups, highlighting the need for early evaluation of such markers, since along with oxidative stress, this pathway is only discriminant in the early days of reperfusion. In these conditions, apoptosis markers could be primarily detected in tubular cells as previously described in human cadaveric kidney allograft [26]. Taken together, these results support that ischemia condition and duration [27] could influence the level and the localisation of the apoptotic process.

Our study investigates the chronological development of early immunity in various preclinical ischemic conditions. Endothelial activation, a prerequisite in the initial recruitment of leukocytes to the site of injury, is a key of innate immunity response initiation commonly assessed by vascular endothelium markers expression such as selectin adhesion molecules. In our conditions, P-selectin mRNA expression was increased early after reperfusion (H3) in CS and WI + CS groups then reached control values at D7. However, WI did not affect this expression. Early immunity could be partially mediated by Damage Associated Molecular Pattern molecules (DAMPs) which bind the TLRs [16,28,29]. These DAMPs are released by cells that have been damaged by IR and thus closely associated to oxidative stress, apoptosis and necrosis [30,31]. TLRs constitute an integral part of the innate immune response and play a pivotal role in IRI, though the role of each receptor is still unclear [32]. The stimulated-TLRs pathway induces the production of pro-inflammatory cytokines such as IL-1β, TNFα, IL-6, IL-8 as well as expression of cell adhesion molecules like VCAM-1 on endothelial cells [33]. In a recent study using deficient TLR4 mice, Pulskens et al. show that TLR4 initiates an exaggerated pro-inflammatory response upon renal IRI and subsequently controls the induction of an innate immune response [17]. In our study, TLR2 expression followed the level of renal injury, particularly after D3. TLR4 overexpression was found in CS and WI + CS, but was not significantly different between both groups. Thus, TLR4 expression appears due to cold ischemic injury while TLR2 is a more discriminant marker of ischemic stress level. MCP-1 was significantly overexpressed in WI + CS from D3 supporting that the degree of injury seems to modulate the MCP-1 response. MCP-1 recruits monocytes and dendritic cells to the sites of tissue injury and inflammation. In addition, DAMPs are known to stimulate IL-1β secretion from the NLRP3 (NOD-like receptors Protein 3) inflammasome inducing pro-inflammatory cytokines production and immune cells invasion [34]. As IL-1β, IL-6 could be produced by macrophages to stimulate immune response in damaged tissue leading to expand inflammation. Interestingly, we observed an IL1Rn mRNA (gene encoded to Interleukin-1 receptor antagonist) increased expression at D3 which could be appearing in response to IL-1β production. We reported an IL-10 mRNA over-expression by high intensity of IRI up to one week after reperfusion. As previously described, IL-10 could be a protecting agent against renal IRI decreasing TNFα expression and reactive oxygen species production [35]. Our observed IL-10 levels could be linked to autologous immune response induced by CS as our results showed no differences between CS and WI + CS. In our results, this alarming environment, stimulated in the WI + CS condition was characterized by an increase of TLR2 and TLR4 mRNA supporting a DAMPs pathway, stimulating an upregulation from D3 of MCP1 and macrophages invasion.

The originality of this study was the graft follow-up during the first three months after transplantation. In accordance to the findings of the first week of reperfusion, we observed a deleterious effect of WI when is combined with CS. Indeed, the difference at D3 in term of renal function in each group was also observed at 3 months. As previously described in our DCD mimicking model [36], associating WI + CS, we observed an inflammatory response characterized by endothelial activation remaining until three months after reperfusion with an increase of MCP-1, and VCAM-1 expressions. These results were supported by lymphocytes and monocytes recruitment already described in this model seven days after reperfusion and maintained 3 months later. As these parameters did not reach statistical differences compared to control in WI or CS groups, these results highlight the direct relationship between the intensity of IR and chronic outcome. Above a certain degree of injury, specific pathological processes are rendered irreversible and will unavoidably lead to chronic failure.

Accordingly, we observed a level of tubular atrophy and fibrosis directly dependent on the severity of ischemia three months before. We underlined that WI alone also promotes fibrosis development although the inflammatory response was not present suggesting a stable process. As expected, CS group exhibited a collagen expression level between WI and WI + CS. Moreover, the development of renal fibrosis is still ongoing in WI + CS group, as indicated by profibrotic pathways activation analysis. While in WI or CS group they appear inhibited or reduced, we showed a trend towards increased for TGFβ and a significant rise of its first downstream mediator pSmad3 in WI + CS, indicate a maintain production of collagen and a progression of renal fibrosis accentuated by MMP-2 decrease expression supporting a less extracellular matrix degradation. In addition, these three parameters are not affecting three months after reperfusion by WI or CS conditions, conversely to BMP-7. In fact, CS group exhibited a high expression of BMP-7 in favour to a reduction of TGFβ pathway activation and an inhibition of collagen synthesis [37]. Plasminogen pathway is also involved in extracellular matrix homeostasis and subsequent fibrosis development [38]. We observed an upper expression of PAI-1 in ischemic groups which attained significant difference in WI + CS group which is associated to a down-expression of tPA compared to WI or CS groups. In these conditions, as BMP-7, the tPA expression is increased related to control value indicating a high extracellular matrix degradation which could explain, in part, the minor intensity of red Sirius staining in these groups.

## Conclusions

In conclusion, the severity of ischemic injuries induced by WI in transplantation process found in kidneys from DCD has a crucial impact in early immune reactivity, graft recovery and outcome, suggesting to intensify follow up in case of DCD organs. Recently, we reported that cold preservation time in DCD conditions is an interesting therapeutic window giving the opportunity to treat the graft without systemic effect especially by pulsatile perfusion, oxygenated preservation or with pharmacological molecules [39]. The complexity of the injury exposed herein strengthens the need for adapted animal models able to evaluate varying degrees of injury and new protocols in clinic-like conditions. Such translational models offer the possibility to study various aspects related to kidney preservation and to develop accurate protocols for organ management from DCD easily applicable in clinical situation.

## Abbreviations

WI: Warm ischemia; CS: Cold storage for 24 h at 4°C; WI + CS: Warm ischemia combined with cold storage for 24 h at 4°C; IRI: Ischemia-reperfusion injury; DCD: Donor deceased after cardiac death; AKI: Acute kidney injury; FeNa+: Fractional excretion of sodium; SOD-1: Superoxide dismutase-1; TGFβ: Transforming growth factor β; pro-MMP-2: Pro-matrix metalloproteinase-2; tPa: Tissue plasminogen activator; MCP-1: Monocyte chemoattractive protein-1; BMP-7: Bone morphogenetic protein-7; PAI-1: Plasminogen activator inhibitor-1; GAPDH: Glyceraldehyde 3-phosphate dehydrogenase; NADPH oxidase: Nicotinamide adenine dinucleotide phosphate oxidase; TLRs: Toll-like receptors; IL-1Rn: The interleukin-1 receptor antagonist; DAMPs: Damage associated molecular pattern molecules; NLRP3: NOD-like receptors protein 3.

## Competing interests

The authors declare that they have no competing interests.

## Authors’ contributions

LR carried out protein and PCR studies and performed the statistical analysis, FF participated in conception and design of this study, acquisition of data, analysis, interpretation, and drafted the manuscript, GS has been involved in revising the manuscript critically for important intellectual content, RT drafted the manuscript and performed the statistical analysis, SL carried out PCR studies, analysis and interpretation, JMG carried out the histochemical and immunohistochemical studies, AV carried out surgery in animals, TH participated in conception and design of this study, acquisition of data, analysis, interpretation, and drafted the manuscript. All authors read and approved the final manuscript.

## Supplementary Material

Additional file 1: Table S1 Primer sequences. Click here for file
