# Supplementary material for: Role of warm ischemia on innate and adaptive responses in a preclinical renal auto-transplanted porcine model
Source: J Transl Med. 2013 May 24;11:129. doi: 10.1186/1479-5876-11-129 (PMC3666894; doi:10.1186/1479-5876-11-129)
Supplement: Additional file 1: Table S1 — Primer sequences. [file 1479-5876-11-129-S1.ppt]

## Slide 1
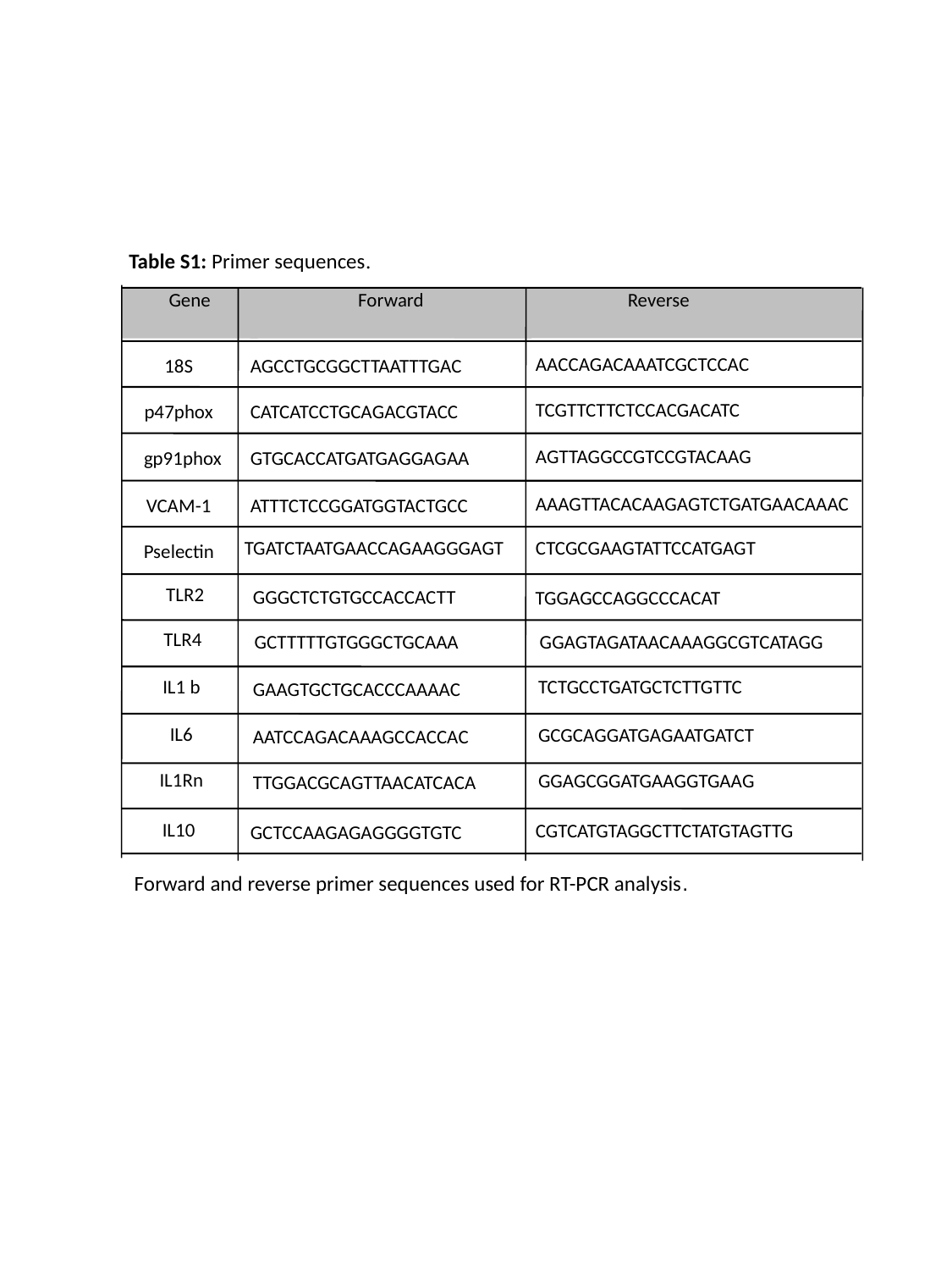

Table S1: Primer sequences.
Gene
Forward
Reverse
AACCAGACAAATCGCTCCAC
18S
AGCCTGCGGCTTAATTTGAC
TCGTTCTTCTCCACGACATC
p47phox
CATCATCCTGCAGACGTACC
AGTTAGGCCGTCCGTACAAG
gp91phox
GTGCACCATGATGAGGAGAA
AAAGTTACACAAGAGTCTGATGAACAAAC
VCAM-1
ATTTCTCCGGATGGTACTGCC
TGATCTAATGAACCAGAAGGGAGT
CTCGCGAAGTATTCCATGAGT
Pselectin
TLR2
GGGCTCTGTGCCACCACTT
TGGAGCCAGGCCCACAT
TLR4
GCTTTTTGTGGGCTGCAAA
GGAGTAGATAACAAAGGCGTCATAGG
IL1 b
TCTGCCTGATGCTCTTGTTC
GAAGTGCTGCACCCAAAAC
IL6
GCGCAGGATGAGAATGATCT
AATCCAGACAAAGCCACCAC
IL1Rn
GGAGCGGATGAAGGTGAAG
TTGGACGCAGTTAACATCACA
IL10
CGTCATGTAGGCTTCTATGTAGTTG
GCTCCAAGAGAGGGGTGTC
Forward and reverse primer sequences used for RT-PCR analysis.
